# Development of a community research link worker role to enable culturally tailored research and empower marginalised communities to participate: the IBISES model

**DOI:** 10.1186/s40900-025-00793-1

**Published:** 2025-10-21

**Authors:** Kate Fryer, Josie Reynolds, Qizhi Huang, Rebecca Mawson, Emma Linton, Habiba Aminu, Johanna White, Ryan Cory, Caroline Mitchell, University of Sheffield, Janet Brown, Fatima Iman-Nabage, Leeds University, Aaishah Aslam, Sheffield Chinese Community Centre, Sarah Ng, Candice Wang, Sheffield African Caribbean Mental Health Association, David Bussue, Sheila Daley, Val Grosset, Carl Case, Lungani Sibanda, Shirley Samuels, Shipshape, Nur Ali, Tanya Basharat, Children and Young People’s Futures, Terezia Rostas, Rosasencis Productions, Rosa Cisneros

**Affiliations:** 1 University of Sheffield, Sheffield, UK; 2 University of Nottingham, Nottingham, UK; 3 South Yorkshire Primary Care Workforce and Training Hub, Sheffield, UK; 4 Keele University, Newcastle-under-Lyme, UK

**Keywords:** Community engagement, Health disparities, Participatory action research, Cultural competency, Research inclusion, Community partnerships, Ethnic minorities, Co-design methodology

## Abstract

**Background:**

People from ethnic minority and socioeconomically deprived backgrounds remain underrepresented in primary healthcare research despite experiencing worse health outcomes and healthcare experiences. Traditional engagement approaches often maintain power imbalances by keeping control within academic institutions, failing to achieve meaningful representation or change.

**Aim:**

This paper describes the iterative development of a model of community engagement across four research projects, aimed at increasing research participation from underserved communities through culturally appropriate co-design and building reciprocal academic-community relationships.

**Methods:**

Using Participatory Action Research methodology, we developed the IBISES model and Community Research Link Worker (CRLW) role through partnerships with voluntary sector organisations serving Black African and African Caribbean, Roma, Chinese, and South Asian communities in South Yorkshire. CRLWs were identified through community organisations, received research training, and joined project teams to lead recruitment, data collection, and support analysis. After each research activity, we conducted debriefing discussions and team meetings to refine the approach.

**Results:**

The CRLW approach was implemented across four studies: a prostate cancer priority-setting project with African &Caribbean men, a contraception research study with women from ethnic minorities, a lung health priority-setting initiative with the Roma community, and a photovoice study examining diverse experiences of aging and dementia services. These projects demonstrated the CRLW model’s effectiveness in accessing traditionally excluded communities, uncovering crucial cultural contexts, and generating meaningful research outputs and community impacts. Through iterative development, we established the IBISES model (Identify community, Build relationships, Investment in training, Support CRLWs, Empower through co-production, Sustain relationships), which provides a framework for implementing the CRLW approach.

**Conclusion:**

The CRLW role enables authentic power-sharing in research, addressing both moral imperatives for inclusion and practical needs for representative evidence. By investing in communities and recognising cultural expertise, this approach moves research engagement toward genuine citizen control and partnership. The IBISES model offers a practical framework for researchers seeking to enhance inclusivity while potentially contributing to greater diversity in academic research careers over time. Further work is needed to explore scalability across different research contexts and evaluate the impact on CRLWs themselves.

## Abstract (plain language summary)

**The problem:**

People from ethnic minority backgrounds and those facing economic hardship are often left out of health research, even though they tend to have worse health outcomes. Traditional research methods don’t meaningfully include these communities.

**The solution:**

Researchers developed a new model called IBISES and created a role called “Community Research Link Workers” (CRLWs). These are people from the communities themselves who are trained to help with research projects.

**How it happened:**

The researchers partnered with community organizations serving Black African, Caribbean, Roma, Chinese, and South Asian communities in South Yorkshire. They identified community members to become CRLWs, trained them in research methods, and made them full members of research teams. These CRLWs then led efforts to recruit participants, collect data, and help analyse results.

**What they found:**

This approach was tested across four different health studies - covering topics like prostate cancer, contraception, lung health, and dementia services. The CRLW model proved effective at reaching communities that are usually excluded from research and uncovered important cultural insights that wouldn’t have emerged otherwise.

**The IBISES framework:**

This was developed into step-by-step approach that researchers could use again: Identify communities, Build relationships, Invest in training, Support the link workers, Empower through working together, and Sustain the relationships over time.

**Why it matters:**

This approach genuinely shares power with communities rather than just consulting them, leading to better research that represents diverse populations. It could also help more people from these communities eventually pursue research careers themselves. The researchers conclude this model offers a practical way for other researchers to make their work more inclusive and representative.

## Background

People from ethnic minority and socio-economically deprived backgrounds are under-represented in primary health and care research [1]. They also have worse health, and worse experiences of using healthcare services [2]. Viewing this problem through the lens of cultural trauma [3], we recognise how dominant oppressive groups have systematically damaged health-protective cultural resources of marginalised communities through their languages, norms, and customs; through institutions like education and healthcare; and through access to material resources necessary for health maintenance.

Part of addressing these health inequities is ensuring adequate representation in primary care research, by addressing the role of institutions and processes in perpetuating inequality in health research [4]. Our research [5] revealed trust as the central issue between underserved communities and academic/healthcare institutions. This trust deficit is deeply rooted in historical and social contexts where communities have experienced exclusion or exploitation in health research.

The cultural competence and values demonstrated by research teams emerged as critical factors in either building or eroding trust. Additionally, practical barriers such as limited technology access and varying literacy levels further constrain participation from underrepresented communities, reflecting broader societal inequalities that manifest in research participation patterns [5].

**Fig. 1:**
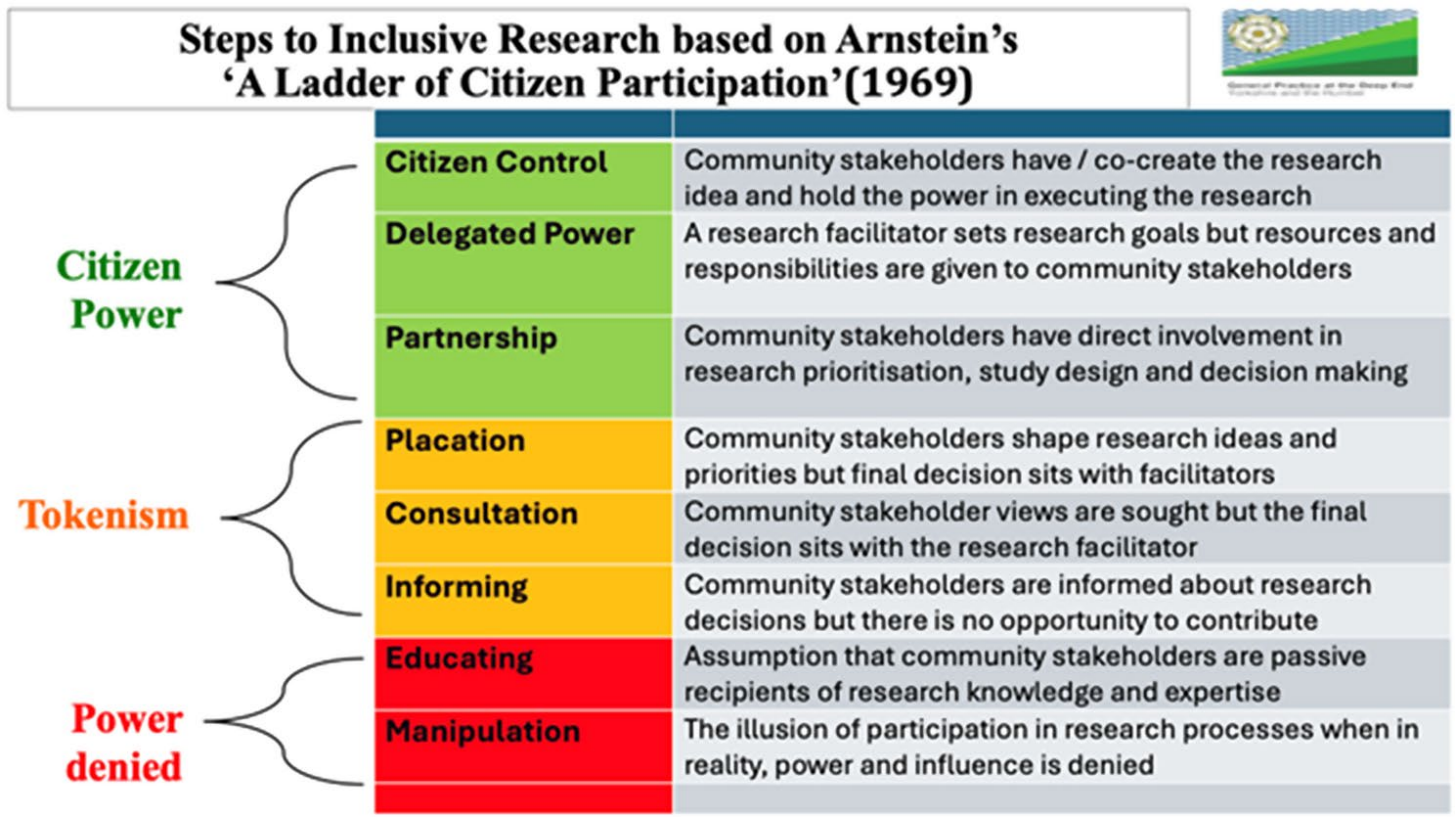
Steps to inclusive research [4]

Traditional attempts to engage with under-served communities in research have often fallen short of any real change, by keeping the power firmly in the hands of academics [4]. In reference to the power ladder shown in Fig. 1, such engagements would be at the bottom of the ladder. We have a moral and ethical responsibility to design studies which aim for the top of this ladder, shifting control of the research agenda through real power sharing. Not only is this a moral and ethical imperative, but our work with communities has suggested that this is the only way to affect real change in research participation. This would in turn lead to greater health equity by ensuring that evidence-based medicine is unbiased and, benefiting wider society when one considers the cost of racism on the NHS and the broader economy [6].

In an ideal world, diversity would be reflected within research teams reducing the hierarchical relationships between academics and underserved communities. Within our own team we have some ethnic diversity and other under-represented backgrounds represented, but not enough. Furthermore, there is multisource evidence that ethnic diversity decreases as seniority increases [7]. Embedding research within communities may impact upon this in the long term, by building capacity and bringing research careers into the spotlight. We are beginning to see this happening locally, with CRLWs pursuing academic studies and taking on academic roles.

The Community Research Link Worker (CRLW) role emerged from our knowledge of other UK-based projects which have used similar roles (e.g. [8]) and the National Institute for Health Research (NIHR) Global Health Research programme which emphasise that international health collaborations require ‘navigating complex dynamics of power and distinct ethical frameworks’.

These roles are often referred in other contexts to as ‘Community Researcher’ or ‘Peer Researcher’ roles. The term CRLW was chosen as it communicates a distinction between academic and community researchers, so that expectations of CRLWs are realistic and allow flexibility in a research role which does not require formal research qualifications. This role complements existing roles within research teams by bringing community specific skills and knowledge into the team.

This role is situated within a context of sustained community engagement, researcher reflexivity regarding power imbalances, and systematic changes throughout the research process. Only through these comprehensive reforms can we achieve the genuine representation necessary for truly equitable health research and ultimately reduce health inequalities across diverse populations.

## Aims

The aims of the Community Research Link Worker Roles are to:

1. Increase awareness of research opportunities within underserved communities
2. Empower community members to take an active role in research processes and the health outcomes of their community
3. Increase research participation by co-designing culturally appropriate research.
4. Co-create long-term reciprocal relationships between academic teams and communities.
5. Increase representation of marginalised communities within research teams through capacity building and exposure to research

This paper describes the iterative development of this role, throughout 4 research projects, into a final model which can be applied by researchers.

## Methods

Participatory Action Research (PAR) is a framework and umbrella term for methodologies which enable co-production of research within an ongoing long-term partnership [9]. It is an approach to research rather than a research method, which seeks to situate power within the research process with those who are most affected by the issue under study [10, 11].

We attempted to embed these principles over the courses of four funded studies. Two of these studies were Patient and Public Involvement (PPI) - also referred to as Community Engagement (CE) - activities, and two were research studies. Having established relationships with local community organisations, we then worked together with those organisations to develop the CRLW role, and the infrastructure to support the role. The community organisations all had charitable status as recognised voluntary care and service organisations (VCSOs) which served Black African and African Caribbean, Roma, Chinese, South Asian communities in South Yorkshire.

CRLWs were identified through various methods including: organisational recommendations from community partners who knew individuals with strong community connections; snowball sampling where existing community networks were used to identify suitable candidates; previous involvement in community health work or research activities; and nomination by community members who recognised individuals with appropriate skills and community standing.

CRLWs met the research protocols at different stages depending on the study design and community readiness. In priority-setting PPI studies, CRLWs were involved from the initial protocol development phase, helping to shape the research questions and methods. In established research studies, CRLWs typically joined during the recruitment and data collection phases, though they were oriented to the full protocol and provided input on culturally appropriate adaptations. This flexible approach ensured meaningful participation while avoiding tokenism, as CRLWs had genuine influence over how protocols were implemented in their communities.

This was an iterative process which developed as we gained experience across the four studies and resulted in an established model and way of working. We followed the PAR “plan/act/reflect cycle”, including debriefing discussions with CRLWs where observational field notes and individual researcher reflections were shared. Further discussions on practical considerations, costs, resources, and the effectiveness of the CRLW model took place in weekly team meetings and consultations with community organisation staff.

## Results

### Case studies

#### Case Study 1: Prostate cancer priority setting

**Project overview**

- **Type:** Patient and Public Involvement (PPI)
- **Lead Researchers:** QH, CM, JB
- **Target Population:** Black men (disproportionately affected by prostate cancer) and their caregivers
- **Aim:** To explore perspectives of prostate cancer in black men and identify research priorities for future research

**Building relationships**

- **Initial contact process:**

A relationship was developed over approximately 12 months with SACMHA (Sheffield African-Caribbean Mental Health Association). KF made initial contact and had several phone calls with staff members. The organisation had mixed previous research experiences and set clear boundaries for collaboration. KF attended an event to meet staff and service users, while simultaneously JR met with the Service Lead (DB) to discuss potential collaboration. This connection was established after JRs online seminar presentation about prostate cancer experiences in Jamaica caught the attention of David, who reached out to discuss research from Trinidad regarding prostate cancer inequalities.

- **Trust-building initiatives:**

DERA and colleagues at the University of Sheffield organised a webinar addressing historical injustices and unethical research that had affected people of African-Caribbean backgrounds. The webinar included an expert in Clinical Trials who explained legislation enacted to ensure safer and fairer clinical trials. Additional face-to-face events with ample opportunity for questions further strengthened the relationship.

- **Key relationship elements:**

Throughout this process, the research team prioritised listening and tailoring their interactions based on SACMHAs recommendations, which proved essential for establishing genuine collaboration.

**Identifying and training CRLWs**

- **Identifying suitable CRLWs:**

The service director DB recommended two existing staff members who already had strong community connections as health champions. An initial meeting with potential CRLWs and the service lead established roles, timeline, and financial arrangements. These details were documented in writing, including pay rates in cash not vouchers and expected working hours within a specified timeframe. A follow-up meeting provided more information about the project and allowed the academic researchers and CRLWs to become acquainted.

- **Training the CRLWss:**

Training consisted of a half-day interactive session covering research types and principles, ethical and data protection issues, and specific skills such as running PPI consultations and focus groups. The aim of the training was to build upon CRLWs’ existing community expertise and literacy skills, recognising that their deep community knowledge and engagement abilities were the primary qualifications needed. While CRLWs developed research-specific skills during the training, the foundation of their effectiveness lay in their established community connections, cultural competence, and communication skills. The training also included project-specific information on prostate cancer to ensure the CRLWs were well-prepared for their roles.

**Project activities**

- **Three PPI meetings with total of 53 attendees**

**Fig. 2:**
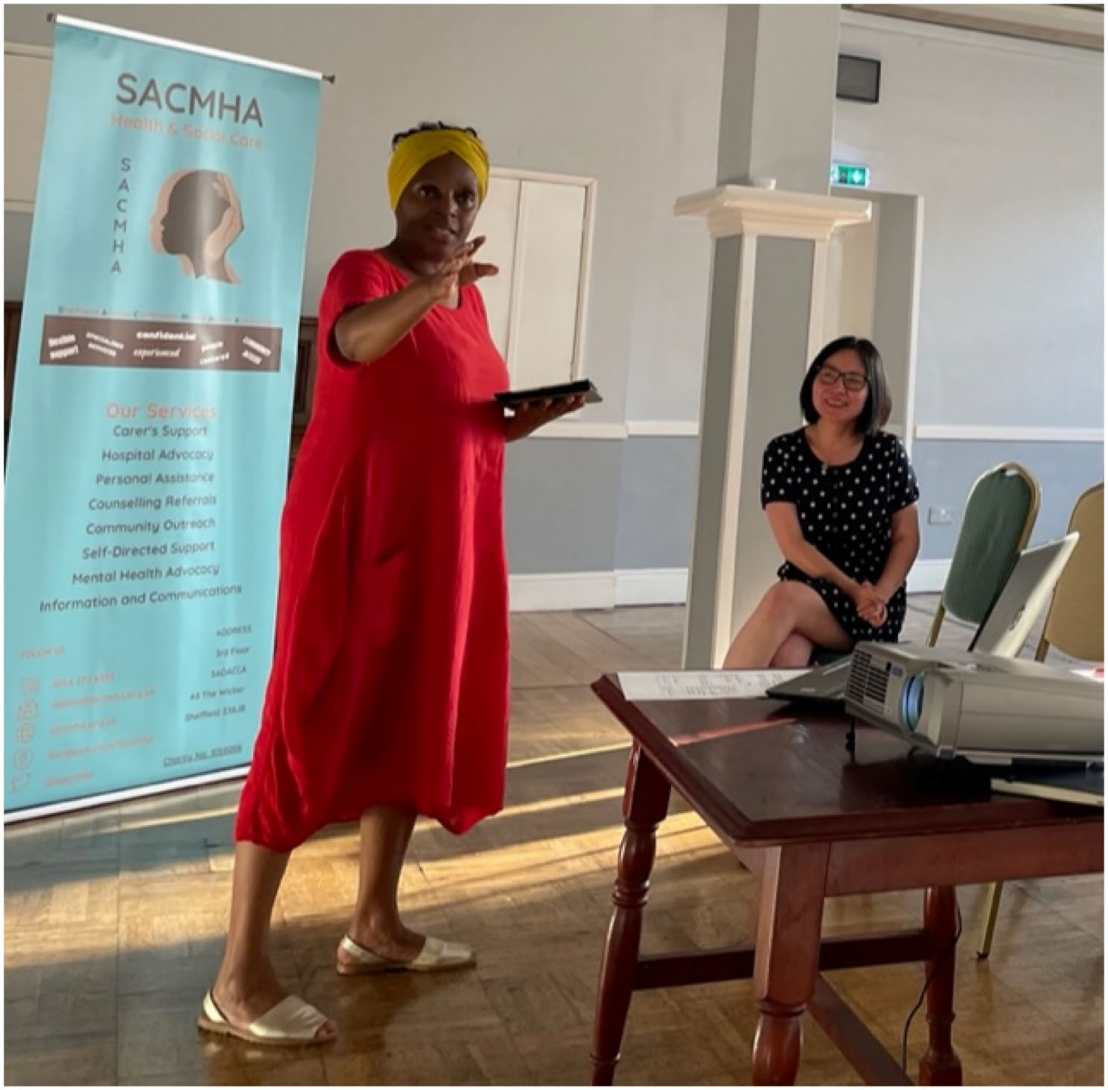
CRLW Val Grosset leading PPI meeting with support from project lead Qizhi Huang

After establishing mutually convenient dates, the CRLWs used multiple recruitment strategies including leveraging their existing community networks, working through SACMHAs service user contacts, utilising faith community connections, and employing word-of-mouth approaches within their established relationships, to recruit participants of African-Caribbean origin with direct experience of prostate cancer. All meetings were held at SACMHA premises with catering provided by SACMHA. Each session was attended by two GPs, two academic researchers, and an oncology consultant (See Fig. 2).

- **Meeting process:**

**Fig. 3:**
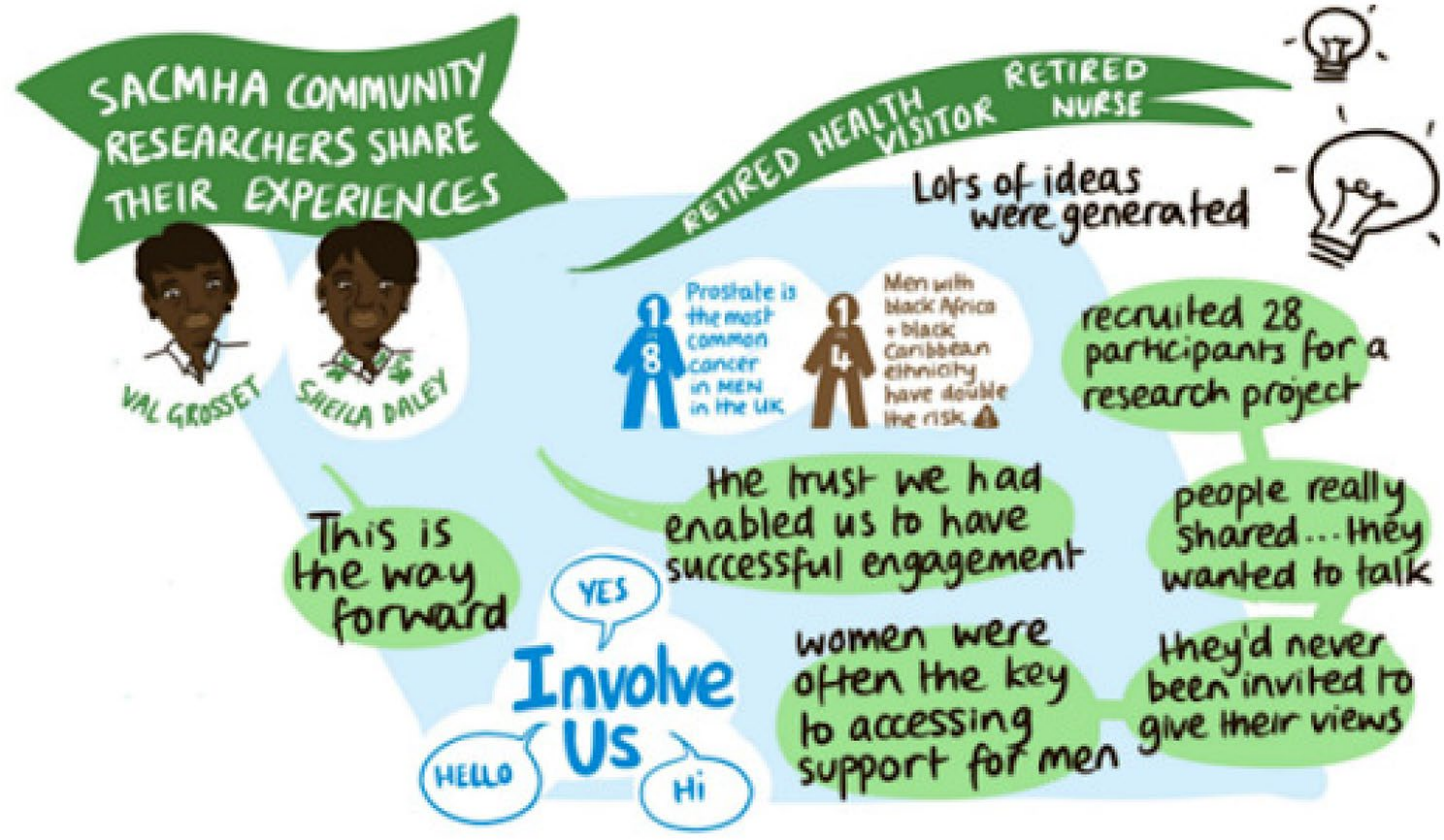
Visual Scribe output from PPI meeting

During the meetings, participants received an overview of prostate cancer and its complications, followed by small group discussions facilitated by the CRLWs. A visual scribe created a summary of the discussions in real-time, while conversations were also audio recorded. Both CRLWs and academic researchers took reflective notes from these recordings, which were then synthesised into a comprehensive summary. After each session there was a debrief with the academic team and the CRLWs (See Fig. 3).

**Outcomes**

- **Conference presentation:**

QH and the CRLWs (VG and SD) presented a poster summarising the priority setting process at the ‘Black in Cancer’ conference in London (2022).

- **Professional advancement:**

QH was appointed to a prostate cancer UK leadership programme and secured two additional grants for work with under-served groups around prostate cancer.

- **Community impact:**

The PPI consultations and JW identified the potential for a dedicated prostate cancer support group. In collaboration with SD, JW secured a 12 month set up grant from Macmillan, and in September 2022 the ‘1in4 Sheffield Prostate Cancer Support Group’ (https://www.1in4spsg.org/) was established as the first African &Caribbean prostate cancer support group in the North of England. This group now supports both PPI activities and qualitative research across multiple projects and grants, as well as the launch of a similar group in Huddersfield.

- **Partnership development:**

The success of this initiative substantially strengthened the partnership between DERA and SACMHA. Study outcomes were shared with participants through multiple channels: direct feedback sessions with PPI participants, presentations at community events hosted by SACMHA, the establishment of the ongoing support group which maintains connection with participants, and distribution of lay summaries. The broader community impact was sustained through SACMHAs continued promotion of the support group and ongoing research opportunities within their service user network.

#### Case Study 2: Understanding women from ethnic minorities’ perspectives about contraception in the UK

**Project overview**

- **Type:** Research study (focus groups and interviews)
- **Lead Researchers:** EL, RM, CM
- **Funder:** Scientific Foundation Board, Royal College of General Practitioners
- **Target Population:** Women aged 16–55 from ethnic minorities
- **Aim:** To understand experiences of contraceptive services, addressing known inequities in provision and outcomes

**Building relationships**

- **Initial public engagement workshop:**

**Fig. 4:**
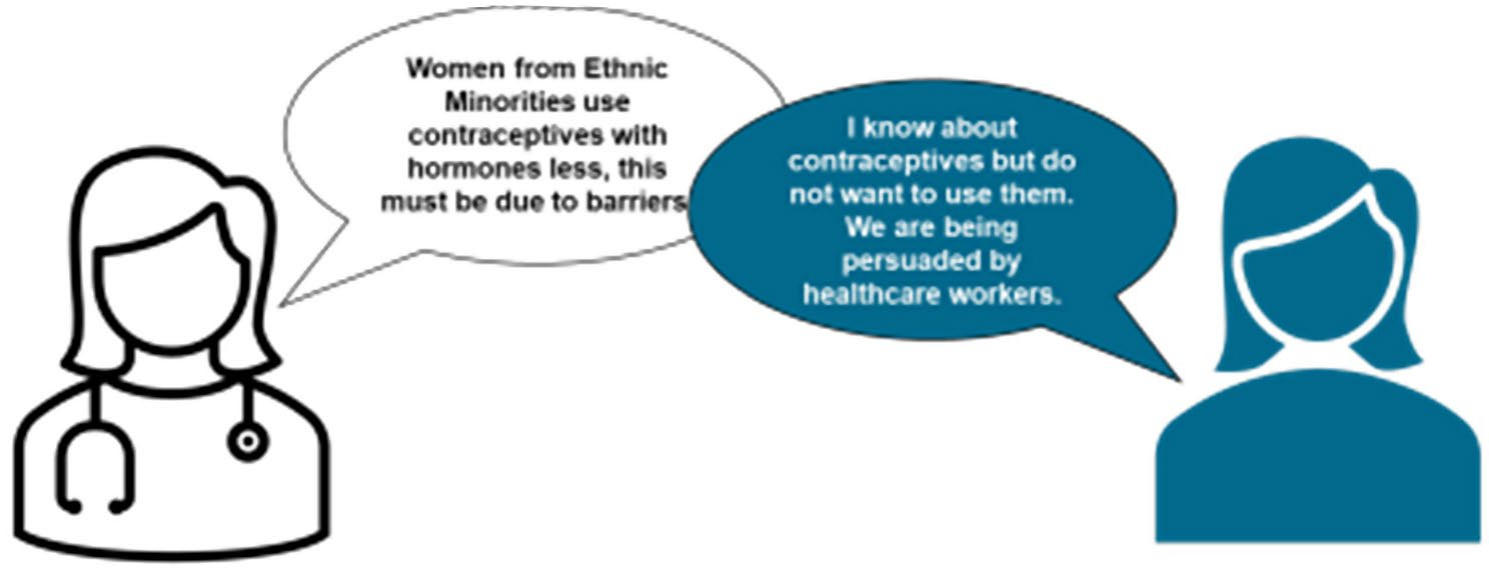
Visual representation of key insight from PPI meeting

The project began with a PPI session that built upon established connections with SACMHA, links to the South Asian community via the Deep End PPI group, and connections with the African community through HA, one of the academic researchers. This workshop drew 20 women participants, including 7 South-Asian, 8 African, and 5 Caribbean attendees. The insights gathered during this initial engagement were instrumental in shaping the focus and direction of the subsequent research project (See Fig. 4).

**Identifying and training CRLWs**

- **Identifying suitable CRLWlink workers:**

Using a role description based on the previous project, two CRLWs were identified from the attendees of the PPI session, with one fluent in two community languages in addition to English. A third CRLW, a student from a Black African community, was known to one of the academic researchers (HA).

- **Training the link workers:**

All CRLWs received comprehensive role descriptions outlining their responsibilities based on the format of the role description used in the prostate cancer project. They completed the standard half-day research training session and received additional project-specific training to prepare them for their roles in the contraception research project.

**Project activities**

- **Recruitment methods:**

The CRLWs utilised their extensive community networks to recruit participants, including contacts from voluntary sector community organisations, faith communities, and English language classes. They successfully recruited 31 women, 24 of whom were born outside the UK, to participate in four focus groups consisting of 6–8 women each.

- **Data collection approach:**

The focus groups were conducted in English, with CRLWs providing language support as needed for participants with limited English proficiency.

- **Analysis process:**

CRLWs received an additional half-day training session on thematic analysis. They were provided with printed, anonymised transcripts of all focus groups and asked to independently identify potential themes. The team then held a joint analysis meeting where CRLWs and DERA researchers shared their notes and worked together to formulate potential themes, which informed the final thematic framework developed by EL and RM.

**Outcomes**

- **Research redirection:**

The findings from the PPI consultation significantly shifted the research focus, as women indicated they were more interested in viewing contraception within the broader context of reproductive health.

- **Outputs:**

The team co-produced a lay output that was shared with all PPI participants and made publicly available (10.15131/shef.data.25259599.v1). The subsequent research highlighted the critical need for culturally congruent consultations and better understanding of individualised care, findings that have been published in a journal article [12].

- **Further projects:**

The project catalysed two additional studies: one exploring contraception side effects specifically for women from ethnic minority backgrounds, and another examining the cultural and societal interplay with contraception side-effects. Notably, one of the lead researchers (RM) supported the student CRLW in successfully applying for a grant to lead research as a stepping stone toward a PhD application.

- **Capacity building:**

The relationships with the CRLWs have continued beyond the project, contributing to building research capacity within these communities.

#### Case Study 3: Lung health priority setting

**Project overview**

- **Type:** Patient and Public Involvement (PPI)
- **Target Population:** Roma community
- **Aim:** To centre the Roma community in NIHR priority research area (lung health) to promote citizen control of the research agenda

**Building relationships**

- **Initial connection:**

The team established a connection to the Roma community through JW, who had previously worked as a Practice Nurse in an area with a high proportion of Roma patients.

- **Challenges encountered:**

Building relationships with this community proved more time-consuming than originally anticipated due to significant marginalisation issues affecting language, culture, and trust. The team relied heavily on two community leaders who already carried substantial workloads, which affected the project timeline. As a result, the project activities were compressed into a shorter timeframe than initially planned, creating additional pressure for the project team.

**Identifying and training CRLWs**

- **Recruitment model:**

The compressed timeline yielded an unexpected benefit by allowing the team to identify two CRLWs who formed a natural mentoring relationship. One more experienced individual acted as a mentor to a less experienced community member, creating an effective capacity-building model. This approach emerged organically and proved valuable for engaging community members who might have been less confident in taking on such a role without support.

- **Training approach:**

Following the established protocol from previous projects, both CRLWs received general research training as well as project-specific training focused on lung health issues.

**Project activities**

- **First meeting process:**

The first meeting included a lay introduction to lung health, followed by a discussion to identify the community’s main concerns. KF documented emerging themes during the conversation, and during a refreshment break, the CRLWs helped translate these themes into the Roma language. Participants then prioritised the themes by placing stars next to the three issues they considered most important, creating a community-validated ranking of concerns.

- **Second meeting process:**

**Fig. 5:**
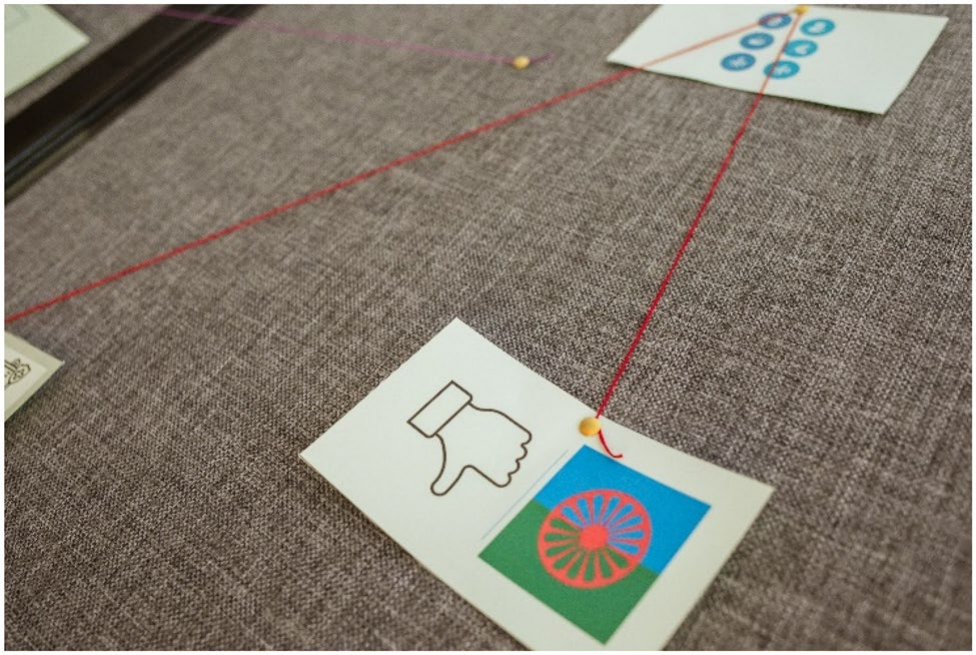
Photo from prioritisation exercise in PPI meeting

Following the initial meeting, the academic researchers conducted a rapid literature review on the prioritised topics. They presented the key findings in an accessible format during the second meeting, which stimulated further discussion and a prioritisation exercise to identify areas where the community felt additional research was needed (See Fig. 5).

- **Meeting logistics:**

Both meetings took place in community venues with food provided by Roma community members. The CRLWs provided essential translation throughout the process, and a digital scribe created visual illustrations of the second meeting to capture the discussions. In total, 14 Roma community members participated in these priority-setting exercises.

**Outcomes**

- **Transformative insight:**

**Fig. 6:**
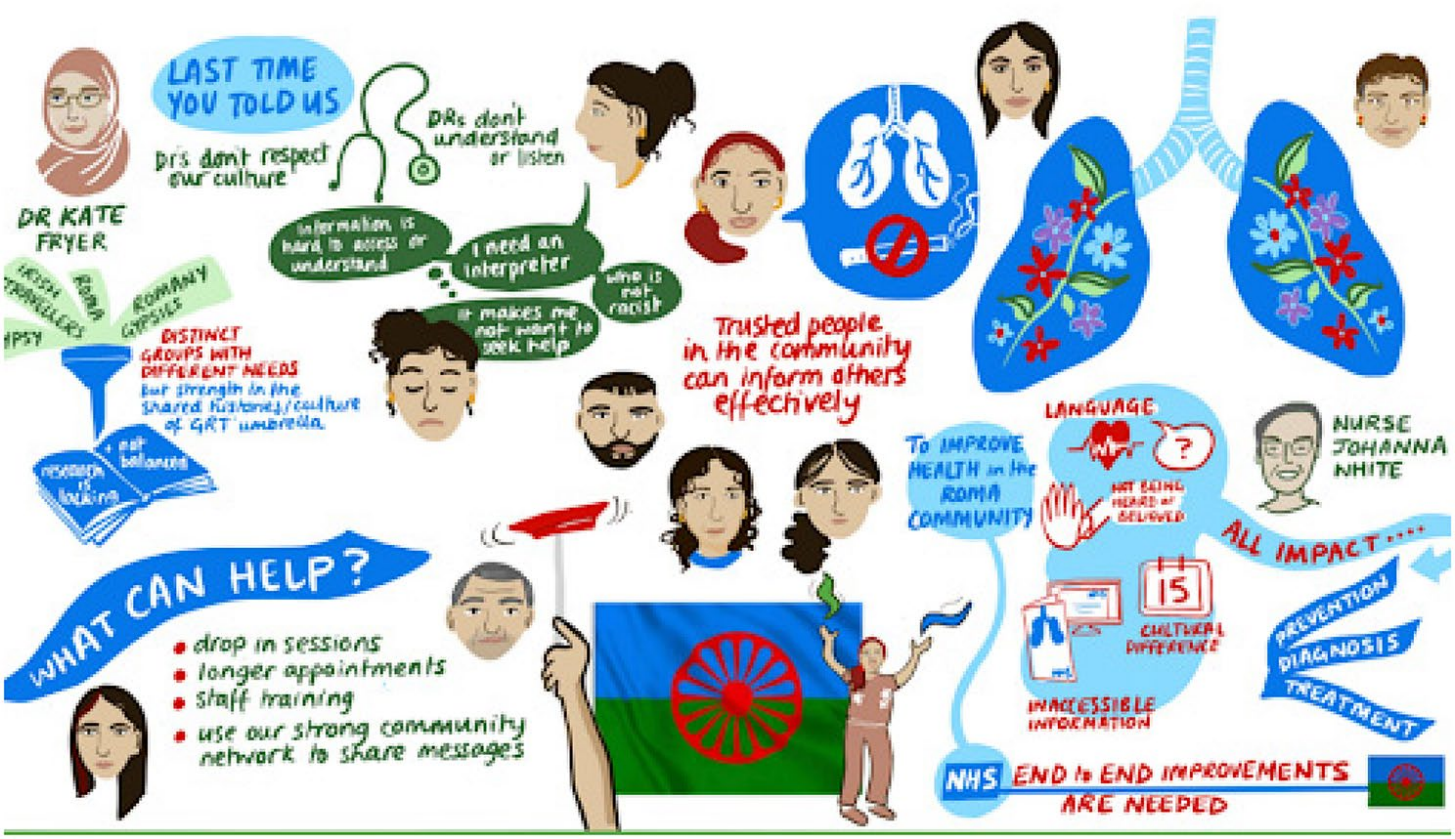
Visual scribe output from PPI meeting

The project revealed insights that transcended the original research focus. Before the team could meaningfully address specific lung health conditions, it became apparent that the Roma community had experienced deep trauma from perceived disrespect of their culture by health professionals (See Fig. 6).

This fundamental discovery necessitated revisiting the original project aims to address these underlying issues first. These insights were subsequently incorporated into future funding applications for work with the Roma community, demonstrating how the CRLW approach can uncover critical contextual factors that might otherwise be missed in conventional research approaches.

#### Case Study 4: Putting me into memory services (PMIMS) study

**Project overview**

- **Type:** Co-produced research study
- **Funder:** Royal College of General Practitioners
- **Target Population:** South Asian, Chinese, and Caribbean communities
- **Aim:** Understand diverse experiences of aging, dementia, and access to memory services
- **Methodology:** Photovoice (visual participatory action research)

**Building relationships**

- **Process inception:**

The PMIMS study originated in 2020 from a conversation between JR and a Dementia Link Worker of South Asian heritage. JR was subsequently invited to attend Sheffield BAME Dementia Link Worker network meetings, which helped develop the initial research concept.

- **Development approach:**

To ensure the study would be relevant and appropriate, the team secured a PPIE grant for extensive consultation with three community groups and visits to housebound elders. These consultations shaped the PMIMS study proposal and led to formal partnerships with three community organisations representing the target communities.

- **Trust-building strategy:**

JR demonstrated commitment to building genuine relationships by attending numerous community events and offering educational talks on various health topics, often unrelated to the PMIMS study. This approach displayed a long-term commitment and genuine interest in the partnership beyond the immediate research objectives, which proved essential for establishing trust with the communities.

**Identifying and training CRLWs**

- **Recruitment process:**

By this point, the CRLW model had become more established and refined through previous studies. Each organisation recruited their own CRLWs with guidance from JR. While the initial plan called for one CRLW from each community, it soon became evident that having two CRLWs working together to share the workload and provide mutual support was more effective. Two communities adopted this approach, while the third had a single, highly experienced community leader and facilitator.

- **Training and support:**

All CRLWs received training in core research skills and study-specific methods from JR and KF. They participated in regular meetings that fostered an open, non-hierarchical atmosphere and had one-on-one check-ins with JR to ensure they felt supported.

The dual role tensions between being community representatives and research team members were addressed through regular supervision sessions where CRLWs could discuss conflicts between community interests and research requirements. We established clear protocols that prioritised community welfare while maintaining research integrity, including mechanisms for CRLWs to advocate for community concerns within the research team. When tensions arose, we held facilitated discussions to find solutions that served both community and research needs, often leading to protocol adaptations that better reflected community priorities.

**Project activities**

- **Recruitment approach:**

The project began by recruiting eight care-partner participants from each community. CRLWs leveraged their networks, including faith-based connections, voluntary sector organisation channels, and word of mouth to share study information. JR supplemented these efforts by distributing information through research networks and community platforms.

- **Orientation process:**

Each community participated in an introductory session at their chosen venue to learn about the study and Photovoice methodology before providing informed consent.

- **Data collection method:**

Following orientation, participants spent eight weeks capturing photos (minimum two weekly), writing captions, and sending materials to JR. They ultimately selected their favourite eight photos to discuss in focus groups.

- **CRLW support role:**

CRLWs provided crucial one-on-one support throughout this process, including language translation, technological assistance, idea development, narrative building, and deadline reminders.

- **Focus group approach:**

The focus groups were jointly facilitated by CRLWs and DERA team members (JR, FK, QH), with language accommodations varying by community: Chinese sessions featured real-time English Cantonese translation, South Asian groups required minimal Urdu translation, and Caribbean discussions were conducted entirely in English. All meetings took place in community venues with culturally appropriate refreshments arranged by CRLWs.

- **Analysis method:**

The analysis process occurred during the second half of the focus group, where KF documented emerging themes from the discussion, and participants actively categorised their photos under these themes using physical photo copies and handwritten theme names on display boards.

**Outcomes**

- **Exhibition impact:**

**Fig. 7:**
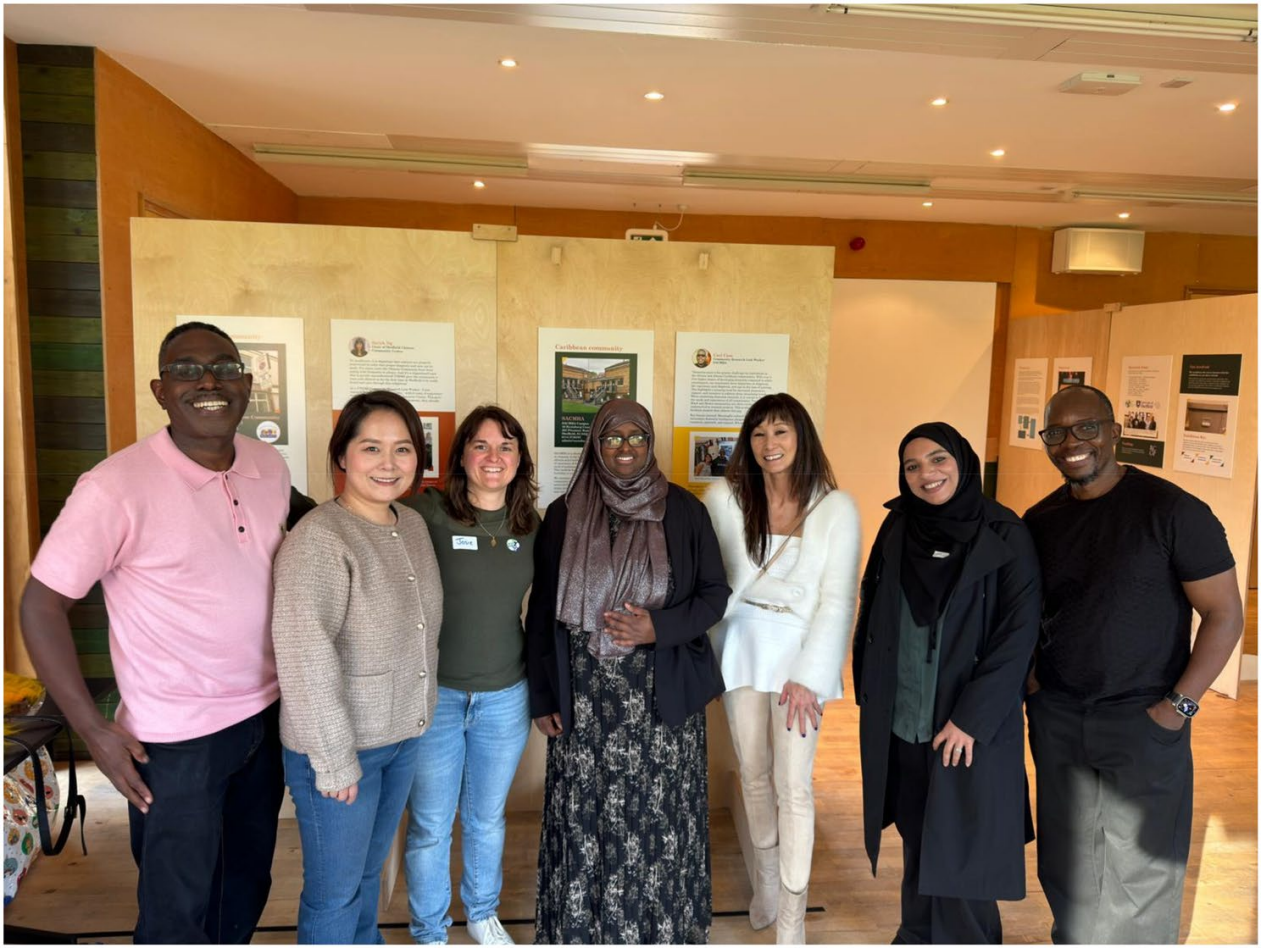
Project lead Josie with CRLWs (left to right: Carl Case, Candice Wang, Josie Reynolds, Sahra Abdi, Sarah Ng, Nur Ali, Lungani Sibanda)

The PMIMS study culminated in a Photovoice exhibition in October 2024, curated collaboratively with CRLWs and creative experts. The exhibition attracted 152 attendees, including policymakers and professionals, and highlighted cultural practices around aging and dementia, community resilience in the face of cognitive decline, barriers to culturally appropriate services, and experiences of structural racism (See Fig. 7).

- **Related events:**

The exhibition was complemented by several events: an opening celebration attended by 62 people featuring cuisine from the Chinese, South Asian, and Caribbean communities; presentations of study findings; a CRLW panel discussion on culturally responsive services; expert talks on culture, ethnicity, and brain health (45 attendees); and a weekend “community take-over” showcasing culturally relevant brain health activities (76 attendees).

- **Policy impact:**

The study’s impact extended beyond the exhibition, with the display featured at Sheffield Council’s Dementia Strategy launch and leading to a partnership that established an Equality, Diversity and Inclusion advisory panel comprised of CRLWs to guide culturally inclusive strategy implementation.

- **Additional outputs:**

Additional outcomes included plans for a virtual exhibition with resources for families from racially minoritised communities and healthcare practitioners. The project has already been featured in the Journal of Dementia Care, with academic publications forthcoming. Furthermore, the study’s findings have inspired a new intervention proposal co-developed by JR and the CRLWs to address identified barriers, which will be pursued as JRs DPhil project.

## Summary

### Summary of four community research link worker (CRLW) studies

The document describes four studies that employed Community Research Link Workers to bridge academic research with underserved communities:

**Participants across all studies**

- Prostate Cancer Priority Setting: 53 African-Caribbean men and caregivers
- Contraception Research: 20 women in initial workshop (7 South-Asian, 8 African, 5 Caribbean) plus 31 women (24 born outside UK) in four focus groups
- Lung Health Project: 14 Roma community members
- PMIMS (Memory Services): 24 care-partners (8 each from South Asian, Chinese, and Caribbean communities) with exhibition events attracting 335 total attendees

**Ethnic Diversity:** The studies collectively engaged African-Caribbean, South Asian, African, Chinese, and Roma communities, with language support provided as needed including English Cantonese translation and Urdu assistance.

**Collective Impact:** These CRLW-facilitated studies resulted in significant outcomes including: establishing the North of England’ first African-Caribbean prostate cancer support group; redirecting contraception research to align with community priorities; uncovering critical insights about healthcare cultural trauma in Roma communities; creating community exhibitions that influenced policy; publishing academic and lay outputs; securing additional research funding; creating pathways for community members to pursue academic careers; and establishing advisory roles for community representatives in healthcare strategy implementation. The approach consistently improved research relevance while building sustained research capacity within underserved communities.

All projects addressed research culture by increasing transparency, inclusion, integrity through open practices, and equality through culturally appropriate research design-demonstrating the effectiveness of the CRLW model in engaging populations traditionally excluded from research.

## The IBISES model of community engagement

**Fig. 8:**
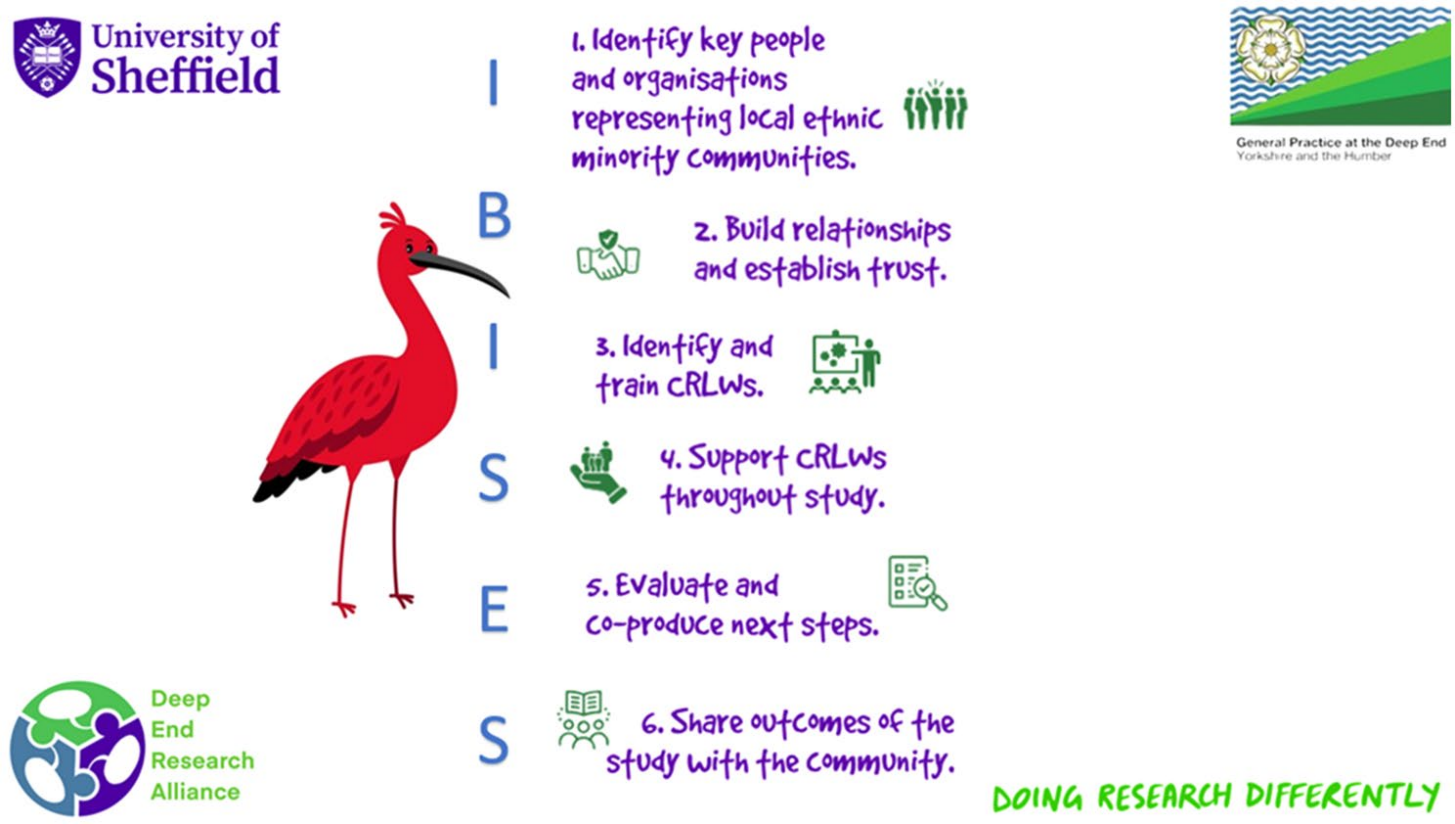
Visual representation of IBISES model of community engagement

The PAR process of plan/act//reflect enabled us to identify the key steps to success across the research projects above, in an iterative process where we were guided by the community. We believe that others may be able to use these steps as a guide to building relationships and undertaking research with underserved communities (See Fig. 8).

Alongside these steps, practical issues need to be considered in terms of operationalising this model, which will be unique to each organisation. These include:

- Identifying the costs associated with using this model, and factoring this into funding applications.
- Ensuring systems are in place to pay partner community organisations, and CRLWs. If possible, arrange for lump sum payment directly to the partner organisation to overcome institutional barriers regarding payments and avoid the need to pay with vouchers.
- Identifying members of the research team who will train and support CRLWs.
- Establishing a written agreement of expectations between the research team and the partner organisation, such as a memorandum of understanding.

The IBISES model should be embedded within a wider PAR process, which ensures that communities are involved in setting priorities for research, and relationships are sustained, the importance of which was emphasised in our previous study [5].

## Discussion

### Summary and comparison with existing literature

The study demonstrated that Community Research Link Workers significantly enhanced recruitment of participants from ethnic minority communities into research and culturally meaningful study design and data interpretation. This represents an important advancement in research methodology for engaging traditionally underrepresented populations, which is particularly important in the light of recent demands by funders to ensure that research is inclusive.

Our IBISES model builds upon and extends previous approaches to addressing underrepresentation in primary healthcare research. While earlier studies have documented the persistent underrepresentation of ethnic minority and socio-economically deprived populations [1], and their poorer health outcomes and healthcare experiences [2], our work moves beyond merely identifying these disparities to implementing practical solutions.

Our approach significantly differs from traditional engagement methods which, as Mitchell et al. [4] critique, often maintain power imbalances by keeping control within academic institutions. Instead, our IBISES model aligns more closely with the upper levels of Mitchell’s power ladder, actively working toward genuine citizen control and power-sharing in research. It does this by investing in communities and therefore mitigating the impact of cultural trauma, which depletes community resources. This represents a crucial advancement from earlier community engagement attempts that remained at the lower rungs of participation.

The CRLW role builds upon similar positions documented in UK-based projects [8] and draws inspiration from the NIHR Global Health Research programme’s emphasis on meaningful community collaboration. However, our model extends these previous approaches by explicitly addressing power dynamics through our choice of terminology and role definition. While similar roles have been termed ‘Community Researcher’ or ‘Peer Researcher’, our specific use of ‘CRLW’ deliberately acknowledges the distinct contributions of community and academic researchers, creating clearer expectations and greater role flexibility, while avoiding hierarchy.

Our work also responds to Bhopal’s [7] findings regarding decreasing ethnic diversity in senior academic positions. The CRLW model provides a potential pathway to address this systemic issue by embedding research within communities and creating new entry points to research careers. This approach aligns with Participatory Action Research (PAR) frameworks [9] but advances them by providing a specific, implementable model for long-term community-academic partnerships.

### Strengths and limitations

This work demonstrated several key strengths in our approach to community engagement through the IBISES model. The methodology successfully taps into existing community networks and activism, particularly valuable given the historical context of community-led responses to systemic exclusion in health and education. By leveraging these established community assets and resources, we were able to create authentic partnerships that went beyond traditional superficial engagement. A significant strength was the establishment of genuine trust between academic teams and communities, facilitated by the CRLWs who served as cultural brokers and bridges between these traditionally separated groups.

However, several limitations must be acknowledged. The relatively small scale and limited geographic scope means that findings may not be immediately generalisable to other contexts or larger-scale implementations. The duration of the projects, while sufficient for model development, may not fully capture the long-term sustainability and impact of the IBISES model. Resource requirements, including training, support, and financial compensation for CLRWs, need careful consideration for future scaling. In regard to the Roma community in particular, we were highly reliant on a small number of key individuals within that community, making it difficult to sustain that partnership in the long term. Additionally, while the model successfully engaged certain community groups, we recognise that some communities may still have remained underrepresented, and further work is needed to understand how to adapt the model for different cultural and social contexts.

Additionally, the effectiveness of the CRLW approach from the community participants’ perspective was not systematically evaluated, as our assessment focused primarily on feedback from community organisation staff rather than capturing direct experiences of community members who participated in the research activities.

### Conclusions: implications for research and practice

Our development of the IBISES model has significant implications for research methodology and practical application in primary care and wider health research. The successful engagement of ethnic minority communities through this approach demonstrates a viable pathway for addressing long-standing underrepresentation in research. The model’s alignment with Participatory Action Research frameworks, while maintaining distinct community-academic roles, provides a practical template for institutions seeking to enhance research inclusivity.

For research practice, our findings emphasise the critical importance of genuine power-sharing and co-production. The IBISES model suggests that meaningful community engagement requires more than traditional recruitment strategies; it demands a fundamental shift in how research teams conceptualise and execute community participation. This has direct implications for how future research projects should be designed, emphasising the need to embed community voices from the earliest stages of research conception through to dissemination.

The model also has broader implications for addressing systemic inequalities in academic research, the IBISES approach could help address the documented decrease in ethnic diversity at senior academic levels [7]. This suggests potential long-term impacts on research workforce diversity and capacity building within underserved communities.

Several key areas warrant further development and investigation:

First, there is a need to explore the scalability of the IBISES model across different research contexts. For example, future work could examine whether the success in community engagement can be maintained when scaled up to larger, multi-site studies, and evaluating the effect of this model on study outcomes/recruitment/representation

Second, we need to evaluate the CRLW role from the perspective of the CRLWs themselves, including the training, support and opportunities they have received throughout the process, and the impact this has had on them and their communities.

These future directions must be pursued while maintaining the core principles that made the initial model successful: authentic power-sharing, respect for community expertise, and commitment to addressing systemic inequities in research participation.

## Data Availability

The datasets used and/or analysed during the current study are available from the corresponding author on reasonable request.

## Abbreviations

UK: United Kingdom
NHS: National Health Service
CRLW: Community Research Link Worker
NIHR: National Institute for Health Research (the UKs largest funder of health and care research, supporting studies to improve people’s health and the NHS).
PAR: Participatory Action Research
VCSO: Voluntary Care and Service Organisation
PPI: Patient and Public Involvement
SACMHA: Sheffield African-Caribbean Mental Health Association
DERA: Deep End Research Alliance (Deep End practices are general practices in areas of high social deprivation, typically serving the most disadvantaged populations with complex health needs and limited resources)
PPIE: Patient and Public Involvement and Engagement
PMIMS: Putting Me Into Memory Services
IBISES: Identify community, Build relationships, Investment in training, Support CRLWs, Empower through co-production, Sustain relationships
